# Mediation effect of anxious attachment on relationship between childhood trauma and suicidal ideation sensitive to psychological pain levels

**DOI:** 10.1192/j.eurpsy.2023.2452

**Published:** 2023-09-22

**Authors:** Hannah Ihme, Philippe Courtet, Nathan Risch, Jonathan Dubois, Raoul Belzeaux, Emilie Olié

**Affiliations:** 1 Institut de Neurosciences de la Timone, UMR 7289, Aix Marseille Université, CNRS, Marseille, France; 2 IGF,Univ. Montpellier, CNRS, INSERM, Montpellier, France; 3 Department of Emergency Psychiatry and Acute Care, Lapeyronie Hospital, CHU Montpellier, Montpellier, France; 4 Pôle Universitaire de Psychiatrie, CHU de Montpellier, Montpellier, France

**Keywords:** attachment, childhood trauma, depression, psychological pain, social pain, suicidal ideation

## Abstract

**Background:**

Childhood trauma (CT), depression, and psychological pain are known predictors of suicidal ideation. Recent literature additionally highlights the importance of the attachment system.

**Methods:**

We aimed to predict suicidal ideation through CT, attachment, and psychological and social pain by using mediation models aiming to predict suicidal ideation through CT (predictor) and attachment (mediator). In the same models, we introduced psychological or social pain as a moderator of the relationship between attachment, CT, and suicidal ideation. We included 161 depressed patients and assessed depression, attachment, CT, suicidal ideation, psychological pain, and social pain.

**Results:**

We found (1) a complete mediating effect of anxious attachment (a_2_b_2_ = 0.0035, CI_95%_ = [0.0010; 0.0069]) on the relationship between CT on suicidal ideation, and (2) a significant complete conditional mediating effect of anxious attachment and psychological pain (index of moderated mediation VAS: 0.0014; CI_95%_ = [0.0002; 0.0032]) but not social pain on the relationship between CT and suicidal ideation. Both models were controlled for history of suicidal attempt, depression severity, and sex.

**Conclusions:**

Our results suggest a developmental profile of suicidal ideation in mood disorder that is characterized by the presence of CT and insecure attachment, especially anxious attachment, that is sensitive to experiences of psychological pain. Nevertheless, we cannot conclude that avoidantly attached individuals do not present the same mechanism, as they may not disclose those ideas.

## Introduction

Suicidal ideation is a preceding risk factor for suicide attempts [1, 2] and death [3]. The presence of suicidal thoughts is common in mood disorders, with a prevalence ranging from 47 to 69% [4, 5], and has a critical impact on prognosis. Consequently, it seems important to understand which factors, such as environmental stressors or trait-dependent variables, are involved in the genesis of suicidal ideation [6, 7]. Recent literature has focused on the importance of childhood trauma (CT) [1, 8, 9] and the neurobehavioral attachment system [10–14] in the suicidal process.

The attachment system is one of the neurobehavioral systems that affect the functioning of the whole organism throughout life, especially in interpersonal stressful situations. Its expression in adulthood is shaped by early interactions with the primary caregiver in childhood [15, 16] and therefore marked by the occurrence of CT. CT is defined as any act or series of acts done or omitted by any person 5 years older than the child that result in harm, potential harm, or imminent danger to the child [17, 18]. Cognitive affective schemata acquired during these early periods of life serve as the basis for interpreting interpersonal relationships later on and thus guide perception, thoughts, and actions [19–21]. A secure attachment is thought to be the result of a responsive and caring environment [22], and is expressed through trust in oneself and others, as well as an inner sense of security. The experience of repetitive frightening and frustrating experiences as typical for CT is linked to an insecure attachment characterized by fear and/or avoidance, mistrust, hopelessness, and pessimism [23, 24].

Avoidant attachment expresses in excessive self-reliance and low interpersonal intimacy. Avoidantly attached individuals tend to deactivate their attachment system in times of stress through suppressing and inhibiting support-seeking tendencies [15]. Anxiously attached individuals in line with a strong desire for closeness tend towards hyperactivation strategies, for example, control, force, or intrusion in order to evoke attention or love from attachment figures [25, 26]. Both attachment strategies might prove insufficient in the face of extremely painful experiences such as loss or exclusion [43] and are effectively linked to higher vulnerability to suicidal ideation [27, 28].

In general, painful experiences (entrapment, isolation) and the associated affective state, psychological pain, are thought to be a central trigger of suicidal ideation [7, 29–32]. Psychological pain refers to an interplay of feelings of shame, guilt, humiliation, dread, fear of “losing oneself,” inner emptiness, confusion, emotional flooding, and social phenomena such as withdrawal or freezing [31–35]. Levels of psychological pain are higher in subjects who present suicidal ideation than in those who do not [36]; and predict suicidal behavior in depressed patients even when controlled for depression severity [37, 38]. Furthermore, suicidal ideation can also be triggered by social pain, the affect experienced at separation and exclusion [39, 40]. Depending on the definition, expected exclusion [41] or merely actual exclusion [42] may be included. While psychological pain can be understood as a “broader construct” incorporating feelings of numbing, humiliation, and coping behavior [35] and must not be tied to the social domain, social pain is distinguishable from psychological pain because it occurs in the social context [42]. As dread of separation is a key component of the attachment system, it has been hypothesized that especially social pain leads to an attachment crisis, in which all coping strategies (distancing as well as approaching) fail, and which then turns to a suicidal crisis [43]. In contrast to psychological pain, social pain can be ethically induced in an experimental setting [44].

Recent literature has already linked CT, insecure attachment, suicidal ideation, and psychological pain in nonclinical settings. In a cohort of 371 Iranian colleague students, CT influenced suicidal ideation directly but also mediated through psychological pain [45]. In 2,259 Chinese students, a mediating effect of psychological pain on the relationship between emotional abuse and suicidal ideation was found [46]. Likewise in a clinical setting, Martins et al. [47] report a direct effect of CT on suicidal ideation in 102 subjects with substance abuse disorder. This effect, however, vanished when the capacity to manage psychological pain was introduced as mediator. Introducing attachment as mediator, Musetti et al. [12] showed a mediating effect of anxious attachment on the effect of traumatic life events on suicidal ideation in a cohort of 950 Italian adults. However, in a clinical population another study failed to show a mediating effect of attachment but found a direct effect of emotional abuse on current suicidal ideation in 96 mood disorder patients [48].

## Objectives

To the best of our knowledge, no study so far has investigated all four concepts, CT, attachment, psychological or social pain, and suicidal ideation. We hypothesize that developmental profiles presenting CT and insecure attachment lead to higher expression of suicidal ideation in mood disorder patients and that this vulnerability is intensified as a function of the experienced intensity of psychological or social pain. We suppose that both types of pain activate deep-rooted attachment-related cognitive schemata and trigger aforementioned attachment strategies [49]. Therefore we introduce social and psychological pain as a moderator, considering it as a trigger for the attachment system [49] and especially its potential to lead to an attachment crisis and subsequently a suicidal crisis [31, 43]. We formulate the following hypotheses:

H1: The relationship between CT and suicidal ideation is mediated by attachment:

1. Insecure attachment (anxious and avoidant) reinforces the effect of CT on suicidal ideation.
2. Secure attachment has a buffering effect on suicidal ideation.
  H2: Psychological pain has a conditional mediating effect on the mediation of CT, attachment, and suicidal ideation, with a mediating effect of attachment sensitive to the level of experienced psychological pain.

H3: Social pain has conditional mediating effect on the mediation of CT, attachment, and suicidal ideation, with a mediating effect of attachment sensitive to the level of experienced social pain.

## Methods

### Participants and setting

We analyzed retrospective data from 161 depressed inpatients recruited in the Academic Hospital of Montpellier, France. Patients were classified into three groups based on their history of suicide attempts: 43 of them had attempted in the last 8 days (*recently*), 52 had attempted suicide at least once in their life (*previously*), and 66 never attempted suicide (*never*). General inclusion criteria were current MDE and age of majority. Patients engaging in substance abuse within the last 6 months, current (hypo)manic or mixed episode, lifetime schizo-affective disorder and/or schizophrenia as well as chronic neurologic pathology were excluded. Due to the primary objective of physical pain, patients on tricyclics or NSSRI were excluded due to possible analgesic effects. Six patients were excluded due to missing data. The inclusion period of the study was from June 2015 to May 2021. A trained and experienced clinician assessed psychopathology using the Mini International Neuropsychiatric Interview (MINI 5.00) and the Structured Clinical Interview for DSM-IV Axis II Disorders (SCID II) for borderline personality disorder.

### Questionnaire data

#### Childhood trauma

CT was assessed in retrospective by the French version of the short Childhood Trauma Questionnaire-Short Form (CTQ-SF; 28 items) [17, 50]. For the CTQ, patients rate the frequency of abusive and neglectful behavior on a 5-point Likert-Scale ranging from 1 (“never true”) to 5 (“very often true”). Items can either be added up to a total trauma score or be grouped into five subtypes of maltreatment: emotional, physical, and sexual abuse and physical and emotional neglect. Physical abuse refers to the intentional harming of the child through physical violence; sexual abuse refers to any sexualised contact between an adult and the child regardless of whether it is done with the child’s consent; and finally, psychological, or emotional abuse refers to any act or speech of demeaning, humiliating or intimidating character from caregiver to child. Physical neglect, on the other hand, refers to domestic situations in which the child’s basic physical needs for food, shelter, clothing, safety, and health care are not met, while emotional neglect refers to caregivers’ failure to meet the child’s basic emotional and psychological needs for love, encouragement, and support. The total trauma score ranges from 25 to 125 and scores for each maltreatment type range from 5 to 25. The scale is widely used; internal consistency ranges from 0.70 to 0.90 and retest reliability from 0.66 to 0.94.

#### Attachment

Attachment was measured by the French version of the Relationship Scales Questionnaire (RSQ) [51, 52]. The RSQ contains 30 items, that load on three factors: avoidance (7 items, e.g. “I find it hard to depend on other people.”), anxiety (5 items, e.g. “I worry that I will be hurt if I allow myself to become too close to others.”), and security (5 items, e.g. “I find it easy to get emotionally close to others.”). Those factors build subdimensions, and for each subdimension, sum scores can be calculated by adding up respective items see results of the factorial analyses in Guédeney et al., 2010). Patients respond on a 5-point Likert scale: (1) *Not at all like me*, (3) *Somewhat like me*, and (5) *Very much like me.* The factor structure of the RSQ demonstrates good psychometric qualities: moderate internal consistency (Cronbach α 0.66 for avoidance factor, 0.69 for anxiety factor, and 0 .60 for security factor) and good interrater reliability (intraclass correlation avoidance factor-ICC = 0.80; anxiety factor-ICC = 0.85; security factor-ICC = 0.78). Patients living alone without partner were asked to respond either for their last relationship experience or for their typical behavior in close relationships.

#### Suicidal ideation

We measured suicidal ideation by the suicidal item of the French version of the Beck Depression Inventory – second edition [53] that reads as follows: (0) *I do not have any thoughts of killing myself.* (1) *I have thoughts of killing myself, but I would not carry them out.* (2) *I would like to kill myself.* (3) *I would like to kill myself if I had the chance.* Patients were asked to choose the response that best described their suicidal ideation status. A previous study showed that a single suicide item from a depression rating scale is a valid approach to assess SI compared with the Scale for Suicide Ideation [54]. This method was previously used in large clinical studies, such as the STAR*D [55] or more recent studies [54, 56, 57].

#### Depression severity

Depression severity was measured by the clinician-rated Inventory for Depressive Symptomatology (IDS-C) [58]. The IDS-C asks the clinician to evaluate the patient’s typical depressive symptoms on 30 items regarding the symptom severity during the last 7 days (e.g. item 6 – *Mood (Irritability):* (0) *Does not feel irritable.* (1) *Feels irritable less than half the time.* (2) *Feels irritable more than half the time.* (3) *Feels extremely irritable virtually all the time.*). The sum score provides information about symptom severity. The English version shows good internal consistency (Cronbach’s α = 0.88) and high external validity (r_HRSD_ = 0.92, r_BDI_ = 0.61).

#### Psychological pain

Psychological pain was evaluated by a visual analog scale, PPP-VAS [37]. Resembling the scales commonly used in the assessment of physical pain [59], PPP-VAS is a well-established tool to measure psychological pain in suicidal cohorts [38]. By means of the PPP-VAS current, mean, and worst psychological pain can be evaluated. Worst psychological pain predicted significantly suicidal events in a prior study [38], therefore we only included worst psychological pain in our statistical analysis. Participants rated the worst intensity of psychological pain during the last 2 weeks on a scale from 0 (none) to 10 (maximum possible pain).

#### Social pain

Social pain was assessed by the Need-Threat Scale (NTS) after the subjects played the Cyberball game. The Cyberball game is a validated paradigm of social exclusion during which participants are instructed that they would play with two other players an online ball game. But instead, they play with a preset computer program and are given a cover story to ensure that they believe the other players are human. The Cyberball game comprised 50 throws with two successive conditions. In the first condition, participants played with the other two players and received the ball as many times as the virtual players (1/3 of the throws). In the second condition, participants were excluded by the two other players during the 20 last throws. Successively, participants filled out the NTS. The NTS assesses 20 subjectively experienced consequences of being excluded during the game, including ratings of self-esteem (“I felt liked”), belongingness (“I felt rejected”), meaningfulness (“I felt invisible”), and control (“I felt powerful”), on a scale ranging from 1 = “not at all” to 5 = “very much”. To create the score of social pain, the total score was reverse-coded (100 – total score). The score ranges between 0 and 80. The higher the score was, the more intense was the perception of social exclusion and social pain.

### Statistical analysis

First, we calculated the Spearman’s Rho correlation coefficients to display shared variance between the metric variables that we wanted to include in the mediation analyses, namely CT, attachment, suicidal ideation, depression, and psychological and social pain.

Then, we calculated multiple mediation analyses. In the first models, we tested the H1, the mediation effect of attachment on the relationship between CT and suicidal ideation by employing the SPSS Macro PROCESS Version 3.5 by Hayes [60], model 4. PROCESS operates on the principle of ordinary least square regressions; confidence intervals and interference statistic are calculated through bootstrapping with 5000 samples.

Second, the hypothesized moderated mediation models (H2 and H3) were tested in separate models using a bootstrapping approach to assess the significance of the indirect effects at differing levels of the moderator [60]. CT served as the predictor variable, with the three factors of attachment as parallel mediators. As in the simple mediation analysis, the outcome variable was suicidal ideation, additionally here, the level of psychological pain and social pain was the proposed moderator. The term conditional indirect effect refers to the fact that path coefficients – effects of the mediator to the outcome variable (b) and the direct effect of the predictor CT on the outcome suicidal ideation (c’) depend on a moderator (shown in Figures 2 and 3). Statistically those coefficients are expressed as a function of the moderator variable and are therefore reported by inserting model values for the moderator. PROCESS calculates the *b* coefficients automatically by inserting 3 values for m: one value marking the 16th (low), 50th (medium), and 64th (high) percentile respectively. Concerning the interference statistic, an *index of moderated mediation* was used to test the significance of the moderated mediation, that is, the difference of the indirect effects across levels of psychological and social pain [60]. The models were calculated using the SPSS Macro PROCESS Version 4.1 by Hayes [60], model 15, with bias-corrected 95% confidence intervals using bootstrapping with 5,000 samples. Significant effects are supported by the absence of zero within the confidence intervals.

For all models, we corrected the standard errors for heteroscedasticity [61], and report unstandardized regression coefficients. Effects were controlled for the severity of depression, the history of suicide attempt (recently, previously, never), and sex, which were included as covariates. In two separate models, we additionally added borderline personality disorder as binary variable as covariate. We calculated models for the CTQ total score and all CT subtypes, but only display in detail the results for CTQ total score in the paper.

## Results

### Description of the cohort

Our cohort was predominantly female (72.5%), single (65.2%), depressed (66.5%), and had a high school diploma (73.3%). 66.5% of patients were diagnosed with a unipolar, 33.5% with a bipolar depression. 23.% of the patients were diagnosed with borderline personality disorder. Sociodemographic and clinical characteristics of the cohort are shown in Table 1.

**Table 1.** Description of socio-demographic and clinical characteristics of the whole sample (*n* = 161)

Sociodemographic variables	*n*	%
Age μ (SD)	37.63	(14.46)
Females	117	72.5
Civil status		
Single	105	65.2
In relationship	56	34.8
Education		
No high school diploma	43	26.7
High school diploma or more	118	73.3
Borderline personality disorder	38	23.5
Bipolar disorder	54	33.5
History of suicide attempts		
Recently (<8 days)	43	26.7
Previously (>1 month)	52	32.3
Never	66	41.0
Self-rating questionnaires	μ	(SD)
BDI total score minus suicidal item	18.17	(6.85)
BDI – suicidal ideation (item G)	1.06	(1.02)
NTS – total social pain score	53.86	(12.0)
PPP-VAS – worst psychological pain during the last 14 days	8.75	(1.75)
CTQ – total trauma score	49.61	(18.78)
CTQ – physical abuse	8.24	(4.74)
CTQ – emotional neglect	13.58	(5.21)
CTQ – physical neglect	8.19	(3.61)
CTQ – sexual abuse	7.07	(3.66)
CTQ – emotional abuse	12.54	(6.25)
RSQ – factor avoidance	21.48	(4.72)
RSQ – factor anxiety	15.39	(4.56)
RSQ – factor security	17.08	(3.88)
Clinician-rated questionnaire		
IDS-C – total depression score	38.53	(8.70)

Abbreviations: BDI, Beck depression inventory; CTQ, childhood trauma questionnaire; IDS-C, clinician-rated Inventory for depressive symptomatology; PPP-VAS, physical psychological pain visual analogical scale; RSQ, relationship scale questionnaire.

**Figure 1.**
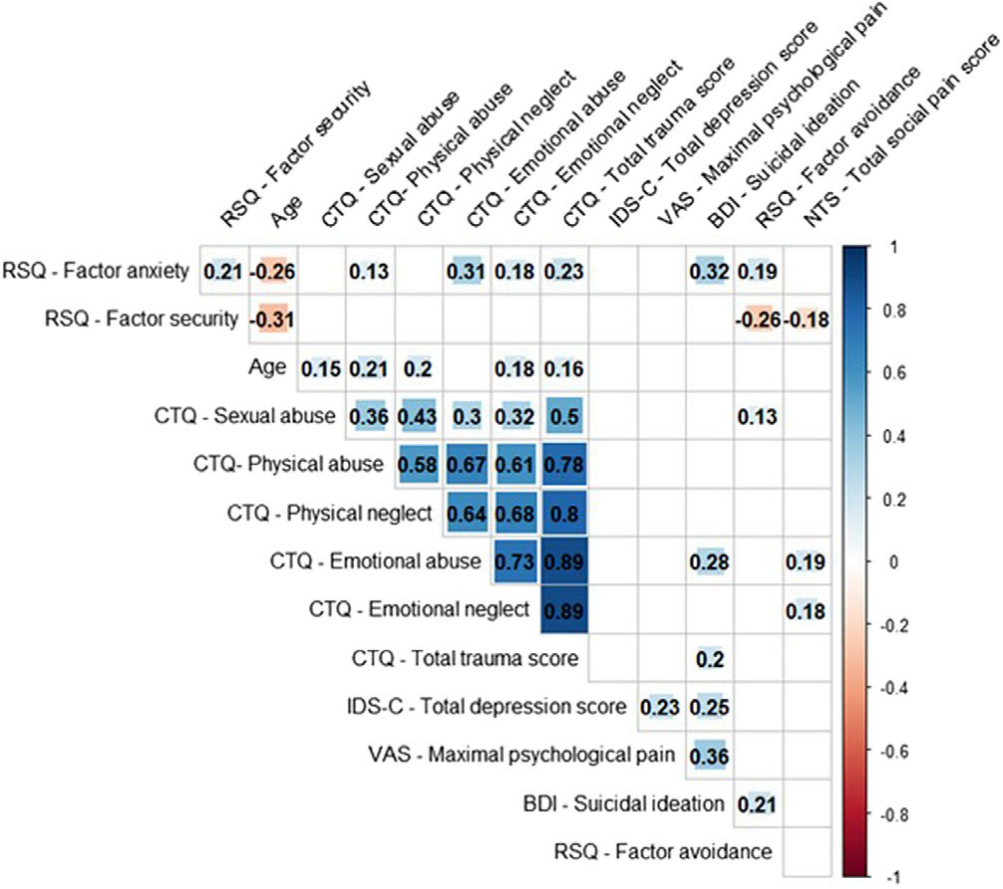
Bivariate Spearman’s Rho correlations, with only significant coefficients being displayed. Strength and direction of the relationship (positive or negative) of the correlation is labeled and additionally indicated by color (negative correlation depicted in red, positive correlation in blue) and size of the square. Correlation coefficients were interpreted according to Cohen’s conventions [62]. A Rho coefficient |ρ| ≥ 0.10 indicates a weak relationship, |ρ| ≥ 0.30 a mediocre relationship, and |ρ| ≥ 0.50 a strong relationship.

Correlation coefficients can be found in Figure 1. Values of the trauma subtypes were highly correlated with each other, security factor presented a mediocre negative correlation with the avoidance factor social pain, and a positive correlation with the anxiety factor of attachment. Further, attachment anxiety was mediocrely correlated to the avoidance factor, the total trauma score, physical neglect, emotional abuse and neglect, and suicidal ideation. Suicidal ideation was mediocrely correlated to psychological pain, depression, total trauma score, and emotional abuse. Last, social pain was mediocrely positively correlated to emotional abuse and the total trauma score, and negatively to the security factor of attachment.

### Mediation analyses

#### H1: Mediation effect of anxious attachment on relationship between CT and suicidal ideation

**Figure 2.**
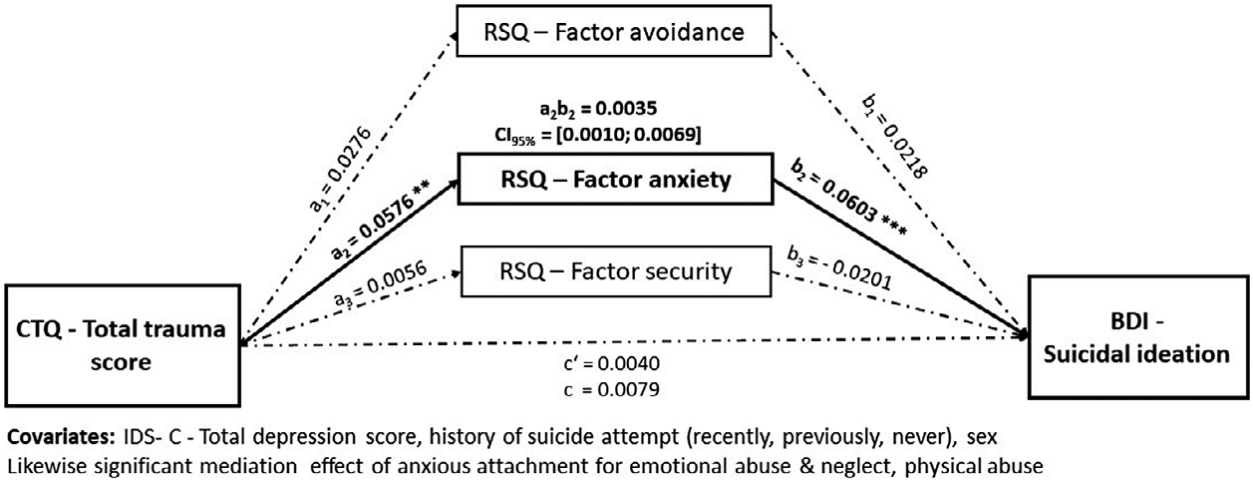
Mediation model on the effect of childhood trauma on suicidal ideation through its effect through three parallel attachment mediators. Regression coefficients are labeled with significant levels *p < 0.05, ** *p* < 0.01, *** *p* < 0.001. Significant paths are in bold.

The first mediation analysis conducted using ordinary least squares path analysis showed that CT (total trauma score) had a complete mediated effect through anxious attachment factor on suicidal ideation a_2_b_2_ = 0.0035, CI_95%_ = [0.0010; 0.0069]. We found neither a direct nor an indirect effect for the avoidant or secure attachment factor (shown in Figure 2). In a second analysis, we calculated the same model for all subtypes of CT and found the same complete indirect effect of anxious attachment factor for emotional abuse and neglect, and physical abuse (shown in Supplementary material).

#### H2: Moderated mediation effect of anxious attachment on relationship between CT and suicidal ideation, with psychological pain as moderator

**Figure 3.**
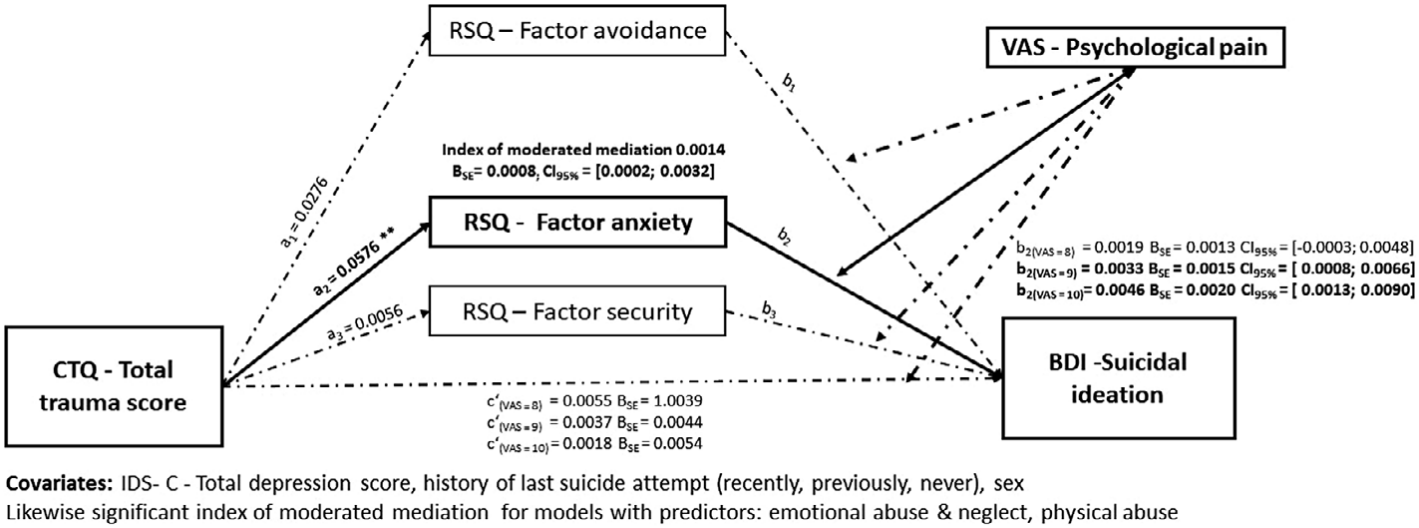
Mediation models on the conditional effect of childhood trauma on suicidal ideation through its effect through three parallel mediators, factors avoidance, anxiety, and security in function of the moderator psychological pain. Regression coefficients are labeled with significant levels *p < 0.05, **p < 0.01, ***p < 0.001. Regression coefficients for b are only displayed for the effect of RSQ-anxiety on suicidal ideation, as it was the only path where significant index of moderate mediation was found. The calculated b and *c*’ coefficients are marked at the side of the diagram. Significant paths are in bold.

Next, we tested the hypothesized moderated mediation model, in which psychological pain moderates the effect of path *b* (shown in Figure 3). Higher CT was associated with higher attachment anxiety, a_2_ = 0.0576, SE = 0.0186, *p* = 0.003. Psychological pain was found to moderate the effect of anxious attachment and suicidal ideation (Unstandardised interaction = 0.0238, SE = .0105, t = 2.2646, *p* = 0.0250). We found a moderate mediation effect of psychological pain on the relationship between CT, attachment anxiety, and suicidal ideation (index of moderated mediation = 0.0014, CI_95%_ = [0.0002;0.0032]). This indicates that individuals with a developmental profile of CT and anxious attachment reported higher suicidal ideation the higher they reported psychological pain levels. The conditional indirect effect was strongest in those reporting the highest psychological pain (64^th^ percentile; effect = 0.0046, SE = 0.0020, 95% CI = [0.0013; 0.0090]) and did not reach significance in those reporting the lowest psychological pain (16^th^ percentile, effect = 0.0019, SE = 0.0013, 95% CI = [−0.0003; 0.0048]). Psychological pain moderated the effect of attachment avoidance on suicidal ideation (Unstandardised interaction = −0.0232, SE = .0111, t = −2.0864, *p* = .0387). However, there was no indication of moderated mediation of psychological pain on the effect of CT over avoidance and security factor on suicidal ideation. Similar results were obtained for separated models of moderated mediation with emotional abuse and neglect, and physical abuse as predictors (shown in Figure 3, and Supplementary material).

Results remained stable even if borderline personality disorder was included as additional covariate (see Supplementary material).

#### H2: No moderated mediation effect of attachment on relationship between CT and suicidal ideation, with social pain as moderator

Last, we tested the hypothesized moderated mediation model, in which we introduced social pain as moderator. No effects were found when the total trauma score served as predictor, and the index of moderated mediation showed a tendency of significance = 0.0002, CI_95%_ = [0;0.0004]. Consecutively, we did not test for any trauma subtype. Results remained stable even if borderline personality disorder was included as additional covariate (see Supplementary material).

## Discussion

Our results support hypotheses H1 and H2 suggesting a heightened vulnerability to suicidal ideation in anxious attachment which is worsened by psychological pain, but insensitive to social pain. Our results on the mediating effect of anxious attachment on the relationship between CT and suicidal ideation replicate results previously published by several research teams [8, 12, 63, 64] but contradict findings from a previous study from our lab [48]. However, to the best of our knowledge this is the first study reporting a moderated mediation effect of attachment and psychological pain on the relationship between CT and suicidal ideation in a clinical setting.

A heightened vulnerability to suicidal ideation in anxious attachment could reflect an imbalanced push-pull dynamic between associative and reflective cognitive processes [65, 66]. In situations of heightened stress, for example, induced through psychological pain or triggered through circumstances that resemble the original context, fast-associative processes guide perception [67, 68], attention, and cognition [23, 69, 70]. In the case of anxious attachment, those associative processes are based on care-related schemata that stem from experiences of inconsistent, impulsive, frightening, or insensitive care [41, 49] and entail a self-perception as helpless, incompetent and dependent [49, 71]. Similar maladaptive personal schemata (e.g. “I am deeply flawed” or “I am a failure”) are suspected as core feature of depressive symptomatology [72], and as the sequalae of traumatic childhood experiences (“cognitive scars” [70]), especially emotional trauma [73]. As our cohort presents depressive symptoms and high levels of emotional trauma those maladaptive schemata are likely to exist in our cohort and might be activated in subjects that indicated a high level of psychological pain.

Further, reflective processes, especially *reflective functioning* – the capacity to understand the behavior of oneself or another as expression of underlying thoughts, beliefs, affective and motivational states [23] – are less accessible under extreme stress [74]. In anxious attachment, this manifests in maladaptive coping strategies that aim to evoke care from others but also amplify the individual’s own distress [71] in a number of ways, including an overestimation of threat, consistent pessimistic believes about their own stress management capacities, low self-worth and overgeneralization of past interpersonal injuries [25, 49, 71]. Heightened suicidal ideation in anxious attachment could thus result from a) the activation of dysfunctional schemata (“cognitive scars”) especially under psychological pain, and b) maladaptive stress-increasing coping strategies. Together and over time, this could aggravate already persisting negative thought spirals that finally accumulate in suicidal ideation.

Thus, our analyses on anxious attachment might have also reached significance due to a higher disclosure of suicidal thoughts in general. A higher disclosure is in coherence with the general orientation towards eliciting support from others that is present in anxious attachment and was found to be associated with clinical anxiety [75, 76]. In contrast, subjects that scored higher on avoidant attachment might not have disclosed ideation even though it was present. Non-disclosure of suicidal ideation is common in depressive cohorts [75, 77] and might be linked in our cohort to increased cognitive disengagement which is a predominant strategy in avoidant attachment. Cognitive disengagement might serve as a protective factor against suicidal ideation in the short term but could increase the risk for suicidal acts in the long term due to isolation and lack of close relationships [43, 48, 79]. Our interpretation of higher disclosure and the activation of maladaptive schemata also seems in line with the fact that the present cohort presents higher values on suicidal ideation and emotional trauma and therefore might be more vulnerable to the here proposed mechanism compared to the cohort of a former study [48]. Further, our cohort presents average levels of secure attachment tendencies. Those did not dampen the effect of CT on suicidal ideation – contrary to our hypothesis - but it might also contribute to the disclosure of suicidal ideation especially in contrast to avoidant attachment.

Last, we did not find a moderating effect of social pain on the aforementioned mediation effect of attachment on CT and suicidal ideation. It is conceivable that our experimental manipulation (Cyberball paradigm) simply missed its mark, for example, because the patients did not engage with it or that hospitalization buffered the effect of the experimental manipulation. Also, social pain may not be “intense” or of large personal valence necessary to activate dysfunctional schemas and suicidal ideation.

### Strengths and limitations of the present study

With 72.7% our cohort presents a high amount of females who are supposedly under higher risk to display suicidal ideation [79]. Even though we tested for heightened sensitivity by including sex as covariate we cannot fully exclude the possibility that the effect found here is not gender biased. Furthermore, the proposed explanation of maladaptive cognitive schemata is well supported by the literature, nevertheless, we did not assess schemata directly. In this vein, recent papers have especially discussed the role of reflective functioning as a trait vulnerability in insecurely attached individuals [74] however no consensus has been found so far [12, 13]. Future studies might further investigate the role of associative schema and reflective processes in combination with other personality traits in predicting suicidal ideation [13].

Further, we need to highlight that we analyze data in cross-sectional way and conclude on a developmental process in retrospect. The gold standard for studying developmental processes is typically long-term, prospective research. Prospective studies provide different figures on CT than retrospective ones [80]. However, it is generally assumed that participants disclose traumatic experiences more readily in retrospect [80]. Furthermore, a negative bias in the recall of autobiographical memory in depression could bias the admission of CT in our depressed cohort. However, a recent meta-analysis reported that the overall effect size of negative recall of explicit memory in depression is small and mostly bound to the emotional valence of experiences [81]. The CTQ was especially created to question about the frequency of abuse and neglect rather than emotional valence to balance out a biased view. Additionally, the CTQ shows good psychometric qualities, is widely used in depressive cohorts, and we correct our statistical model for depression severity. We therefore believe that CT was realistically captured and that the association we report reflects a real association in the cohort.

The described study is part of a bigger study during which physical pain through thermal stimuli was also assessed. The latter was assessed in a counterbalanced way before or after the Cyberball game, which could have impacted social pain ratings. We did not control for any such effect. Furthermore, we did not control for any medications. It might be possible that medical treatment influenced the perception of psychological pain. Last, we did not investigate intent of suicidal ideation. Suicidal ideation is more common and luckily only a fraction of those with ideation pass from idea to act [82].

## Conclusion

This study suggests a developmental profile of suicidal ideation in mood disorder that is characterized by the presence of CT and insecure attachment, especially anxious attachment, that is sensitive to experiences of psychological pain. Nevertheless, we cannot conclude that avoidantly attached individuals do not present the same mechanism, as they may not disclose those ideas. Future research should therefore focus on a detailed assessment of attachment, dysfunctional cognitive schemata, and reflective functioning.

## Supporting information

Ihme et al. supplementary material Ihme et al. supplementary material

Ihme et al. supplementary material Ihme et al. supplementary material

## Data Availability

Information is available on request.

## Supplementary material

The supplementary material for this article can be found at https://doi.org/10.1192/j.eurpsy.2023.2452.
