# Supplementary material for: Mediation effect of anxious attachment on relationship between childhood trauma and suicidal ideation sensitive to psychological pain levels
Source: Eur Psychiatry. 2023 Sep 22;66(1):e79. doi: 10.1192/j.eurpsy.2023.2452 (PMC10594339; doi:10.1192/j.eurpsy.2023.2452)
Supplement: Ihme et al. supplementary material [file S0924933823024525sup001.docx]

SUPPLEMENTARY MATERIAL
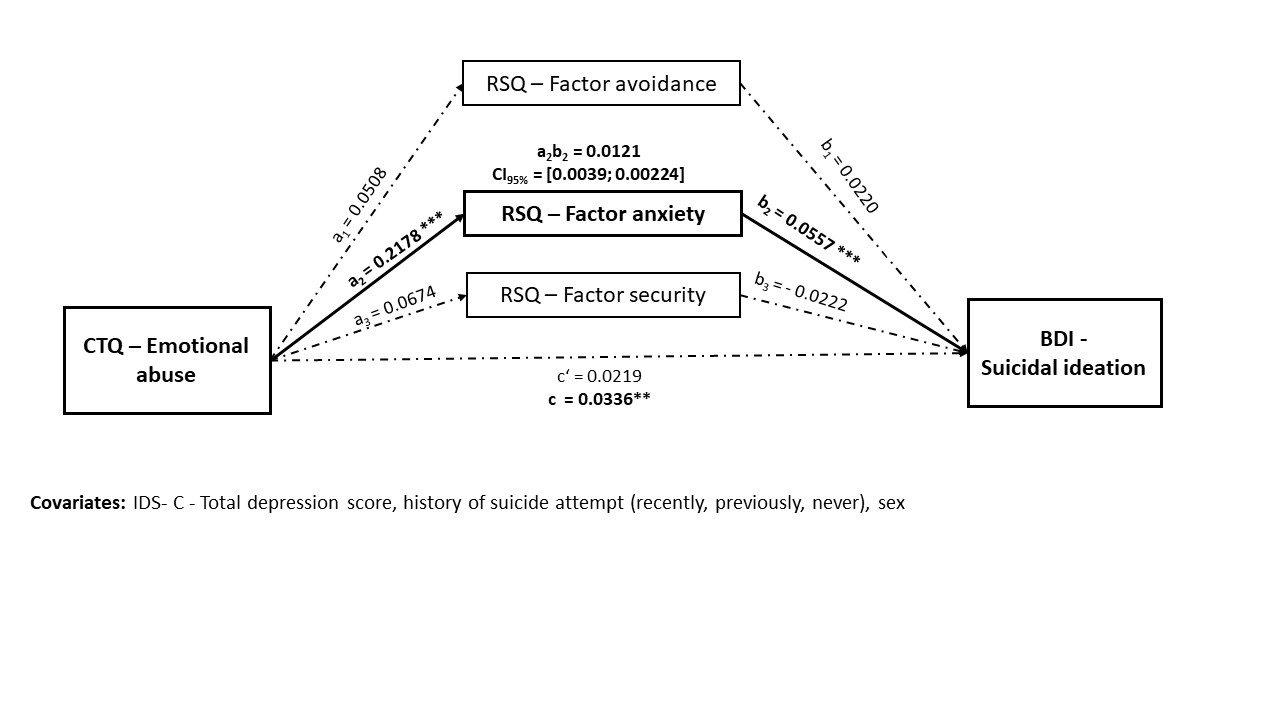

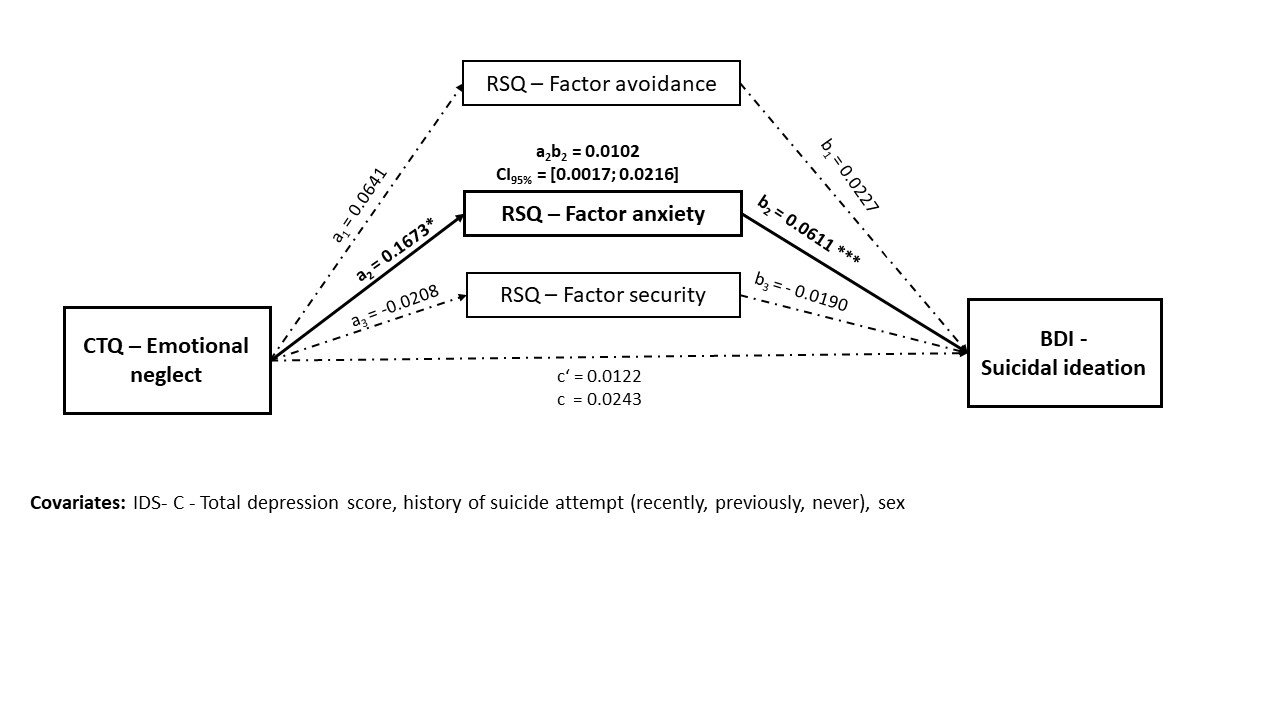


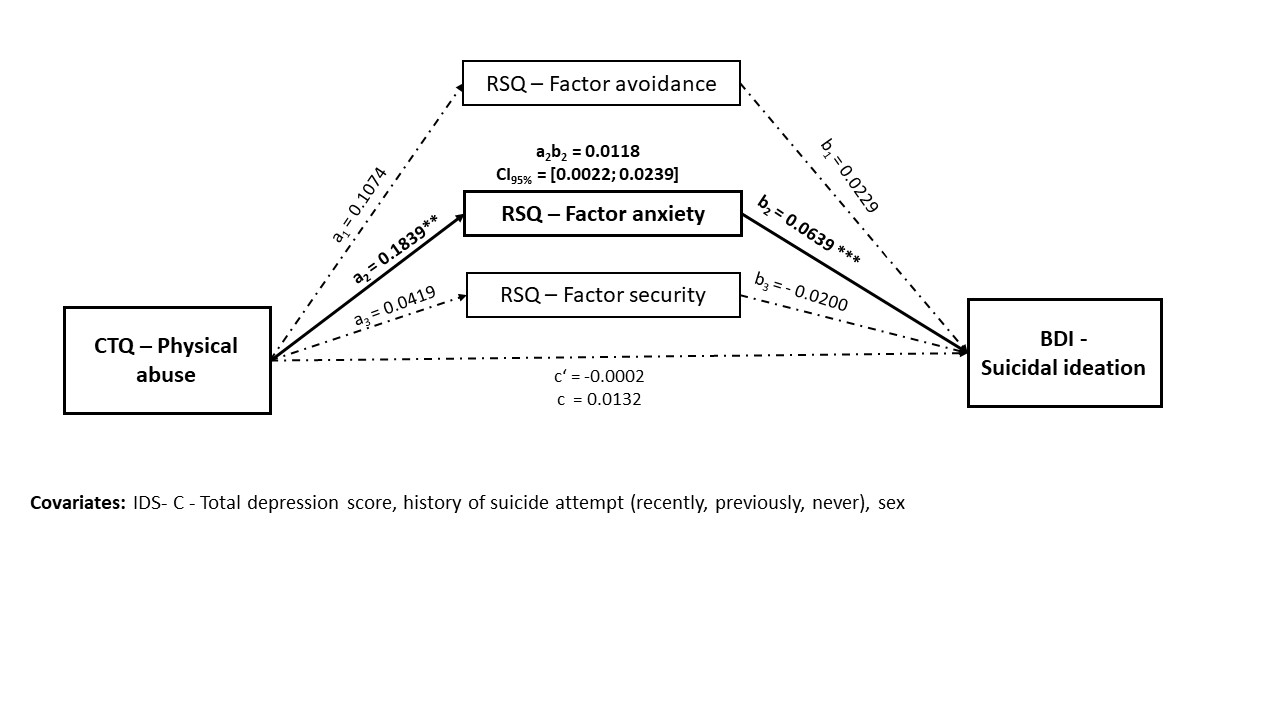


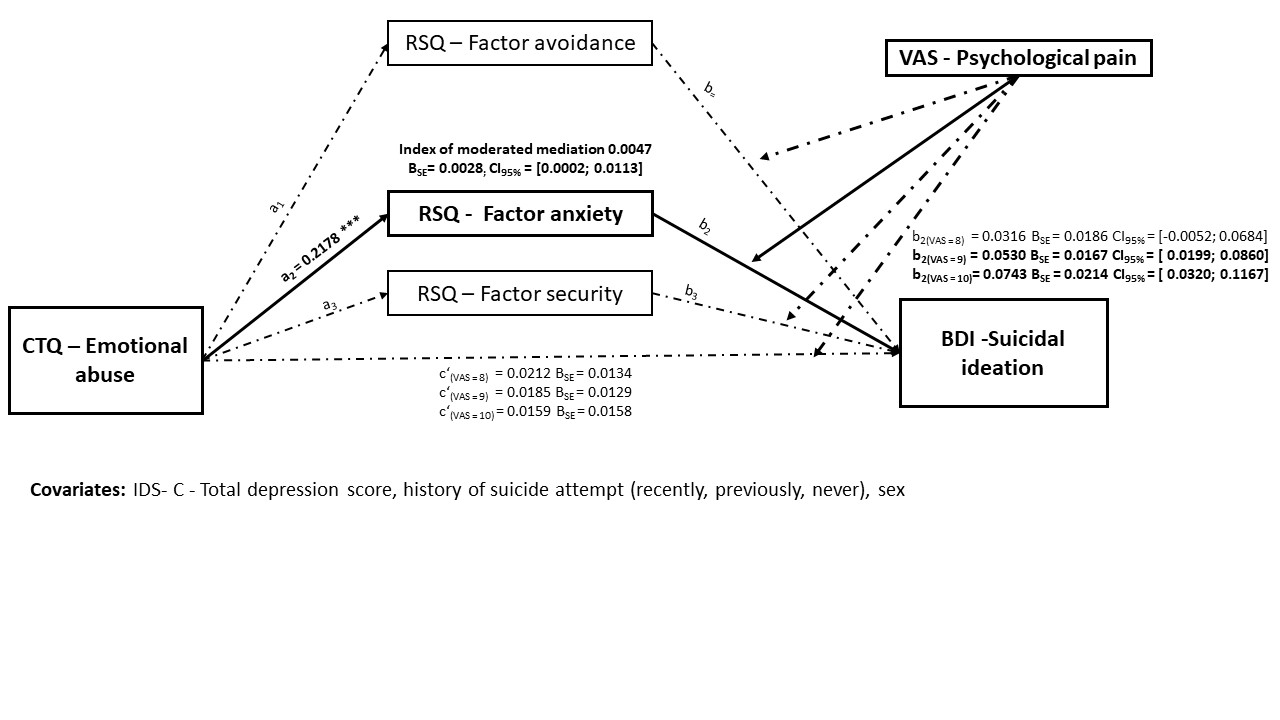

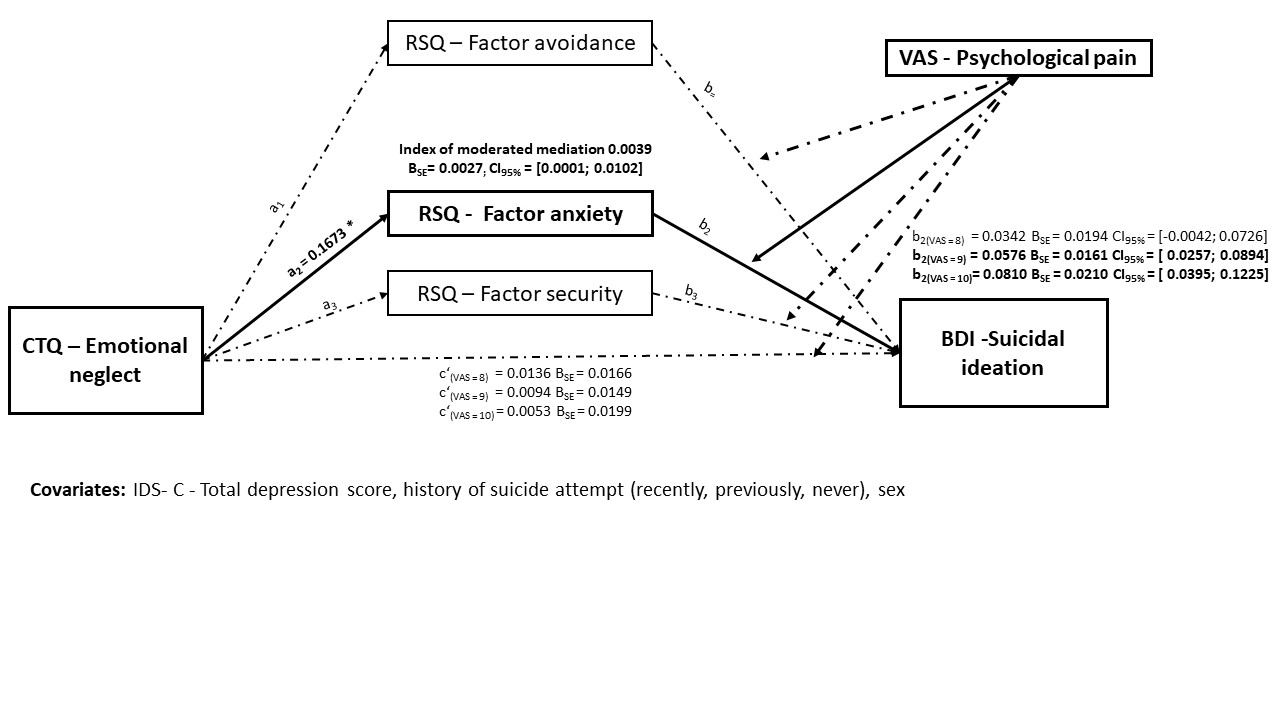

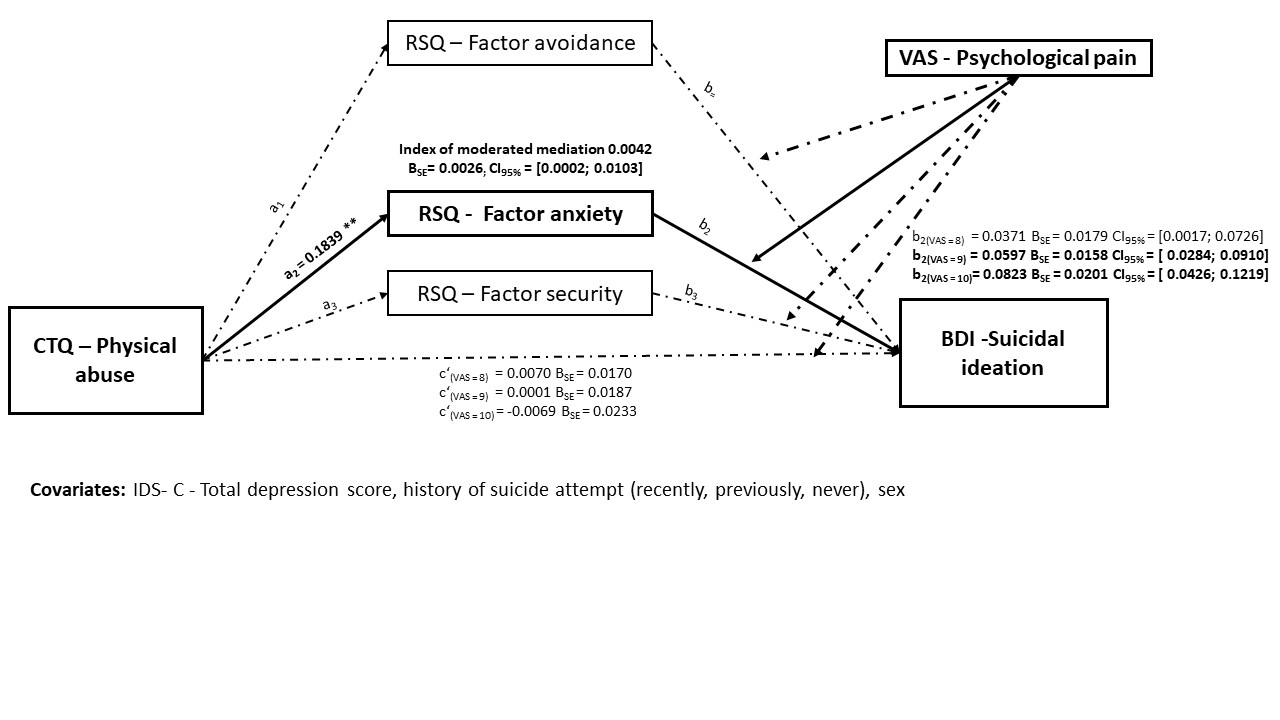


SUPPLEMENTARY MATERIAL BORDERLINE


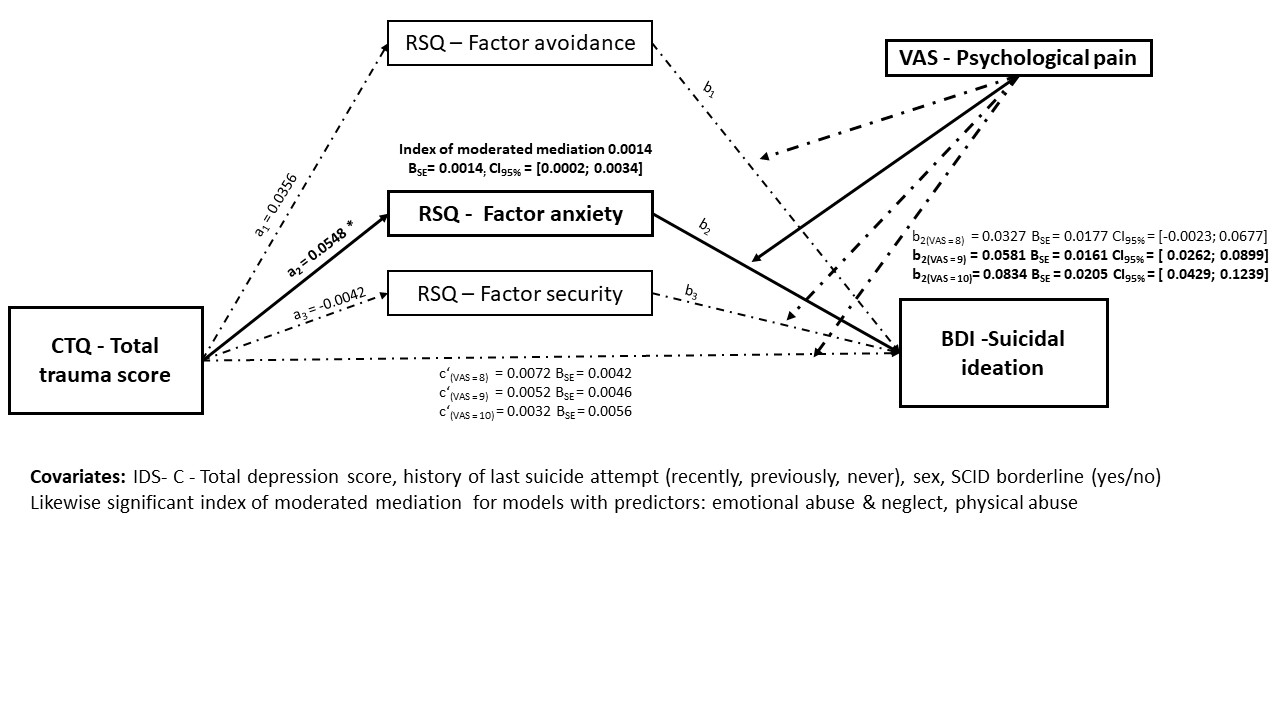


There was no indication of moderated mediation of social pain on the effect of CT over avoidance and security factor on suicidal ideation.

**No moderated mediation effect of attachment on relationship between CT and suicidal ideation, with social pain as moderator.** No effects were found when the total trauma score served as predictor, and the index of moderated mediation showed a tendency of significance = 0.0002, CI_95%_ = [0;0.0004]. Consecutively, we did not test for any trauma subtype.

| *Table SUPPLEMENTARY MATERIAL : Mean and SD of full cohort, patients with Borderline characteristic, and those without, and results of Mann-Whitney-U test comparing Borderline vs Non-Borderline* | | | | | | | | |
| --- | --- | --- | --- | --- | --- | --- | --- | --- |
|  | Cohort  N = 161 | | Borderline  N = 38 | | Non-Borderline  N = 123 | |  |  |
|  | M | SD | M | SD | M | SD | U | *p* |
| BDI - Item G - Suicidal ideation | 1.07 | 1.019 | 1.00 | 0.900 | 1.09 | 1.056 | 2296.50 | 0.864 |
| CTQ – Physical abuse | 8.24 | 4.744 | 10.11 | 5.922 | 7.66 | 4.178 | 1704.50 | 0.007 |
| CTQ – Emotional neglect | 13.58 | 5.210 | 15.08 | 5.509 | 13.11 | 5.048 | 1834.00 | 0.045 |
| CTQ – Physical neglect | 8.19 | 3.613 | 9.74 | 3.881 | 7.71 | 3.401 | 1544.00 | 0.001 |
| CTQ – Sexual abuse | 7.07 | 3.663 | 7.37 | 3.823 | 6.98 | 3.622 | 2179.00 | 0.468 |
| CTQ – Rmotional abuse | 12.54 | 6.259 | 14.84 | 6.533 | 11.83 | 6.023 | 1715.50 | 0.013 |
| CTQ – Total trauma score | 49.61 | 18.783 | 57.13 | 19.968 | 47.28 | 17.852 | 1650.00 | 0.006 |
| VAS-PPP - Worst psychological pain | 8.75 | 1.750 | 9.16 | 1.053 | 8.63 | 1.901 | 2035.00 | 0.204 |
| IDS – Total score depression | 38.53 | 8.696 | 38.87 | 9.715 | 38.43 | 8.396 | 2295.00 | 0.867 |
| RSQ – Avoidance factor | 21.48 | 4.718 | 20.58 | 4.984 | 21.76 | 4.619 | 1994.50 | 0.172 |
| RSQ – Anxiety factor | 15.39 | 4.561 | 16.26 | 4.385 | 15.11 | 4.597 | 1999.50 | 0.178 |
| RSQ – Security factor | 17.08 | 3.875 | 18.53 | 3.674 | 16.63 | 3.840 | 1690.00 | 0.010 |
|  | 37 |  | 123 |  |  |  |  |  |
| NTS – Total Score social pain | 53.86 | 12.875 | 52.62 | 13.54 | 54.26 | 11.55 | 2068.00 | 0.401 |
|  |  |  |  |  |  |  |  |  |
